# Physical activity among women of low socioeconomic status living with HIV in two major cities of Brazil and Mozambique: A cross-sectional comparative study

**DOI:** 10.6061/clinics/2020/e1771

**Published:** 2020-08-21

**Authors:** Lucília Mangona, Iedda Almeida Brasil, Juliana Pereira Borges, Antonio Prista, Paulo Farinatti

**Affiliations:** I Laboratorio de Atividade Fisica e Promocao da Saude (LABSAU), Instituto de Educacao Fisica e Desportos, Universidade do Estado do Rio de Janeiro, Rio de Janeiro, RJ, BR.; II Universidade Pedagogica de Mozambique, Maputo, Mozambique.

**Keywords:** AIDS, Energy Expenditure, Accelerometry, Cardiovascular Risk, Health

## Abstract

**OBJECTIVES:**

Physical activity (PA) may reduce cardiovascular risk and preserve functional capacity of people living with human immunodeficiency virus (HIV). However, only limited research objectively measured PA in patients with low socioeconomic status (SES) in low-income countries, particularly in South America and sub-Saharan Africa. This study compared the PA assessed by accelerometers in women of low SES living with HIV under common antiretroviral therapy (cART) from two major cities in Brazil (Rio de Janeiro, n=33; 40.1±6.1 years) and Mozambique (Maputo, n=50; 38.8±8.7 years).

**METHODS:**

Eligible women wore triaxial accelerometers during seven consecutive days, to estimate their habitual PA and daily energy expenditure.

**RESULTS:**

The proportion of participants with overweight/obesity was greater in Rio than Maputo (57% *vs*. 30%; *p*=0.021), as well as those classified as sedentary based on steps/day (45% *vs.* 22%; *p*=0.02). Sedentary time was prevalent (Median±IQD: Rio-1236±142 *vs.* Maputo-1192±135 min/day; *p*=0.15). Time spent in PA was short, but Brazilians exhibited lower amount of light (111±56 *vs.* 145±51 min/day; *p*<0.001) and moderate-to-vigorous PA (88±3 *vs.* 64±36 min/day; *p*=0.001) *vs.* Mozambicans. The proportion of patients performing 60 min/day of moderate-to-vigorous PA were 58% (Rio) and 82% (Maputo), respectively. Despite of this, estimated daily energy expenditure was equivalent in both groups (1976±579 *vs.* 1933±492 kcal; *p*=0.731).

**CONCLUSIONS:**

Women with low SES living with HIV in Maputo were more active *vs.* patients from Rio de Janeiro. Albeit sedentary behavior was prevalent, the proportion of patients complying with the minimum recommended PA for health was higher than values usually reported in developed countries.

## INTRODUCTION

In 2017, there were approximately 36.9 million people living with Human Immunodeficiency Virus (PLHIV) worldwide, the vast majority in low-income countries (1). A few decades ago, although the survival of PLHIV was low, treatment with a combination of antiretroviral therapy (cART) increased their life expectancy to levels currently approaching the general population (2). However, the long-term effects of viral infection combined with cART toxicities increased the rates of metabolic abnormalities, reduced functional capacity, and chronic diseases among PLHIV, therefore compromising their health and quality of life (3,4). Moreover, HIV/AIDS is a disease embedded in social inequity, affecting those of lower socioeconomic status (SES) at a disproportionately high rate (5,6). Although the universalization of cART allowed greater access to treatment, the exposure of PLHIV with low SES to risk factors, predisposing to disability and chronic diseases is greater in comparison with the wealthier groups (5-7). Therefore, multiple physiological symptoms associated with the HIV and cART are majorly due to poverty (6,7).

Prior reports have warned about the deleterious effects of HIV and cART upon physical and mental functioning, making the preservation of regular employment difficult (5,8-10). PLHIV with low SES often rely on the informal sector to work in jobs with high physical demands, and losing physical capacity may directly affect their opportunities of subsistence (8,10). Additionally, gender inequities are long-standing, and SES is a key factor in determining the quality of life for women, their children, and families (9). A majority of women living with HIV are found in the informal sector, lack legal protection and have increased vulnerability to poverty (10,11). Therefore, the maintenance of work capacity in this group seems paramount, particularly in the low-income countries.

Accumulated evidence indicates adequate levels of physical activity (PA) may help in dealing with the direct effects of HIV and toxic side effects of cART (3,12-16). In fact, low levels of PA are associated with poor physical function in PLHIV (17,18). Therefore, strategies to foster the enrollment of patients in regular PA are often conveyed to preserve their health and overall physical capacity (12). A better understanding of PA levels in PLHIV is crucial in clinical behavioral health programs to manage strategies to improve PA-related health outcomes (4,12). However, systematic reviews and meta-analyses indicate limited studies quantifying PA in PLHIV with discrepant results (16,19). Moreover, there are several barriers precluding the engagement of PLHIV in regular PA. An interesting study (20) has recently summarized 45 quantitative trials on correlation of PA in PLHIV. As expected, their participation in PA relied on a range of complex factors, such as side effects of cART, poor immunity, depression, body pain, occurrence of opportunistic infections, and low SES (8,19). This latter aspect seems to be particularly relevant in low-income countries, given the proportion of citizens classified in this category. SES is a strong and reliable predictor of health outcomes and a determinant of health-related behaviors (5), including PA (18,21).

In short, PLHIV are more likely to be of low SES, with individuals in low SES being more inactive (18,21). Despite this, data on PA among PLHIV are scarce in regions with high concentration of low-income countries and high prevalence of the disease, as South America or sub-Saharan Africa. For instance, in the abovementioned correlates study by Vancampfort et al. (20), only five trials were held in these subcontinents. Moreover, none of the works reviewed in their study and in a further meta-analysis by the same group (19), specifically investigated patients with low SES. Briefly, while the largest public health impacts of sedentary behavior in PLHIV probably occur in low-income countries and among patients with low SES, there is an evident lack of investigations on their PA. Comparative studies including PLHIV with low SES in regions neglected by research might contribute to the current knowledge by showing similarities and differences of PA patterns, as well as providing parameters for comparisons with developed countries.

An additional aspect refers to the use of objective techniques to assess PA in PLHIV. The need to measure the physical work *in situ* favored the development of strategies to avoid biases arising from self-reports. One of the devices most used is the accelerometer, considered the gold standard for PA assessment. Of the 24 studies included in a prior meta-analysis (19), PA was objectively assessed (accelerometers or pedometers) in nine trials, with only two in Africa (within the same study, control and experimental groups) and none in South America. In the correlates study by Vancampfort et al. (19), of the 45 reviewed trials, two applied accelerometers (in USA) and two quantified PA through doubly labeled water (in UK), while none in Africa or South America. Briefly, there is an evident mismatch between where objective assessment of PA has been held and where it is more necessary.

Given these gaps in the literature, the present study compared the PA level through accelerometry in cohorts of low SES women living with HIV and under cART, from urban areas of two major cities in South America (Rio de Janeiro, Brazil) and sub-Saharan Africa (Maputo, Mozambique).

## MATERIALS AND METHODS

### Participants

In a cross-sectional design, we enrolled 83 women of low SES living with HIV. There were 33 from Rio de Janeiro, Brazil (40.1±6.1 years, range 28-53 years) and 50 from Maputo, Mozambique (38.8±8.7 years, range 25-60 years), both groups being followed at tertiary hospitals. Patients were asymptomatic, free of opportunistic infections, and under cART for at least 6 months. The exclusion criteria were cerebral toxoplasmosis or any infectious diseases compromising the central nervous system, and cardiovascular, respiratory, bone, muscle, or joint problems limiting physical function. Average earnings ranged from 1 to 3 minimum wages, corresponding to 6 to 18 dollars/day in Brazil and 2 to 6 dollars/day in Mozambique, which is compatible with low SES according to the World Bank criteria (22). All women lived in peripheral and socially vulnerable areas of the two cities and depended upon public transportation for occupational and leisure activities.

### Experimental design

In both Brazil and Mozambique, the recruitment occurred at ambulatory units of the University Hospitals from August 2017 to December 2018. All female patients attending medical consultations at a tertiary hospital from the University of Rio de Janeiro State (Rio de Janeiro) and at the Polana Caniço Health Research and Training Center (Maputo) were invited to participate in the study, being referred to a researcher who explained the project in detail.

Subsequently, body mass and height were evaluated by means of a mechanical scale with precision of 100 g (Cambé^TM^, Rolândia, SP, Brazil) and stadiometer (Sanny^TM^, São Paulo, SP, Brazil), respectively. After satisfying the eligibility criteria, the patients underwent body mass (scale Tanita^TM^ BF350, Tokio, Japan) and height (SECA^TM^ digital stadiometer, Berlin, Germany) assessments. In both countries, after the anthropometric assessments, the patients wore triaxial accelerometers (ACL) during seven consecutive days, to estimate their habitual PA and daily energy expenditure.

### Objective assessment of physical activity

The PA levels were quantified using triaxial ACL (ActiGraph^TM^ GT3X+6.12.11, ActiGraph^TM^ Corporation, Pensacola, FL, USA). These devices are designed to provide ‘counts’ related to PA in different axes of the body (vertical, antero-posterior, and mid-lateral), and use an algorithm to account the age, body mass, height, and sex. Actigraph ACL quantify the amount of movements performed over a given period with a dynamic range of +/- 8 G-force, and is widely used to measure sedentary time, PA, energy expenditure, and sleep behavior (23). Good relationships of energy expenditure quantified with ACL *vs.* doubly labeled water have been reported (24), including in HIV-infected patients aged 18- to 60 years (25).

Patients were instructed to wear the accelerometer on the hip (23) for seven days during all waking hours, except when bathing or swimming, or in case of allergy or rain (26). Moreover, they had to record the days of wearing the monitor. ACL data were screened for wear time using standard methods (27,28). Daily wear time was determined by subtracting non-wear time from 24h. For a registration day to be valid, a period of at least 10h containing records every 60 sec was required (27,28). For data based on vertical axis counts, non-wear time was defined as ≥60 consecutive minutes with zero ACL counts, allowing up to 2 min with limited movement (<100 counts/min). Consecutive hours with 0 records were considered invalid (29). Data were collected at a frequency of 30 Hz and analyzed in epochs of 60 sec. Data extraction was performed using the Actilife^TM^ software version 6.12.11 (ActiGraph^TM^ Corporation, Pensacola, FL, USA).

Cut-off points for classifying PA intensity using counts/min were previously configured, as follows (27): sedentary (0-99 counts/min), light (100-2,019 counts/min), moderate (2,020-5998 counts/min), vigorous (>5,999 counts/min). Steps/day and MVPA/day were determined by averaging the steps taken per day and time spent per day in moderate- to vigorous PA (MVPA) across valid wear day. The classification of PA from daily steps used categories previously proposed for adults (30) was sedentary (<10,000 steps/day); moderately active (10,000-15,000 steps/day); and active (>15,000 steps/day). Classification of daily MVPA complied with the American College of Sports Medicine (ACSM) recommendations (31) as sedentary (<30 min/day); moderately active (30-60 min/day); active (>60 min/day).

Total daily energy expenditure (kcal/day) was calculated by means of an equation previously used in studies with HIV-infected patients (25) as [average MET x (0.0035 x body mass (kg) x 1440) x 5]. In addition, a value of 1 MET was assigned during non-wear time and when the vertical axis or triaxial counts were below the threshold for sedentary behavior (100 counts/min and 200 counts/min, respectively) (28). Immobility corresponded to periods during which no movement was recorded by the ACL in any of the three axes.

### Statistical analysis

A violation of normal distribution was detected by the Kolmogorov-Smirnov test in regards to ACL data, which were therefore presented as median (interquartile difference-IQD). Age, body mass, height, and body mass index (BMI) had normal distribution, and comparisons across groups were performed by means of independent t-tests. The Mann-Whitney test was applied to compare PA levels between groups. Chi-square tests compared the proportions of patients with overweight or obesity, and the time spent in intensity levels (steps/day and MVPA/day) between patients living in Rio de Janeiro and Maputo. All calculations were performed using the SPSS^TM^ software version 22.0 (IBM, Armonk, NY, USA) and statistical significance was fixed at *p*≤0.05.

### Ethics

All volunteers provided informed written consent before participation in the study. The present study complied with recommendations laid in the Helsinki Declaration and gained approval from local Institutional Review Boards (Brazil: CAAE 96892518.80000.2559; Mozambique: National Bioethics Committee for Health ref: 180/CNBS/11).

## RESULTS

Table 1 presents characteristics of patients recruited in Rio de Janeiro and Maputo. The two samples were similar in age and height. However, body mass, BMI, and relative number of patients with overweight or obesity were higher among Brazilian participants.

Table 2 depicts ACL data in both samples. Immobility and sedentary time were similar in patients from Rio *vs.* Maputo, while time spent in light or moderate activities and overall steps/day were significant greater in patients living in Maputo. Time spent in vigorous activities was very short in both groups, with statistically significant results in patients from Maputo. In consequence, the median time spent in MVPA among patients living in Maputo was significantly greater *vs.* those dwelling in Rio de Janeiro. Despite these differences, the estimated amount of kilocalories spent per day was equivalent in samples from the two countries.

**Figure 1:**
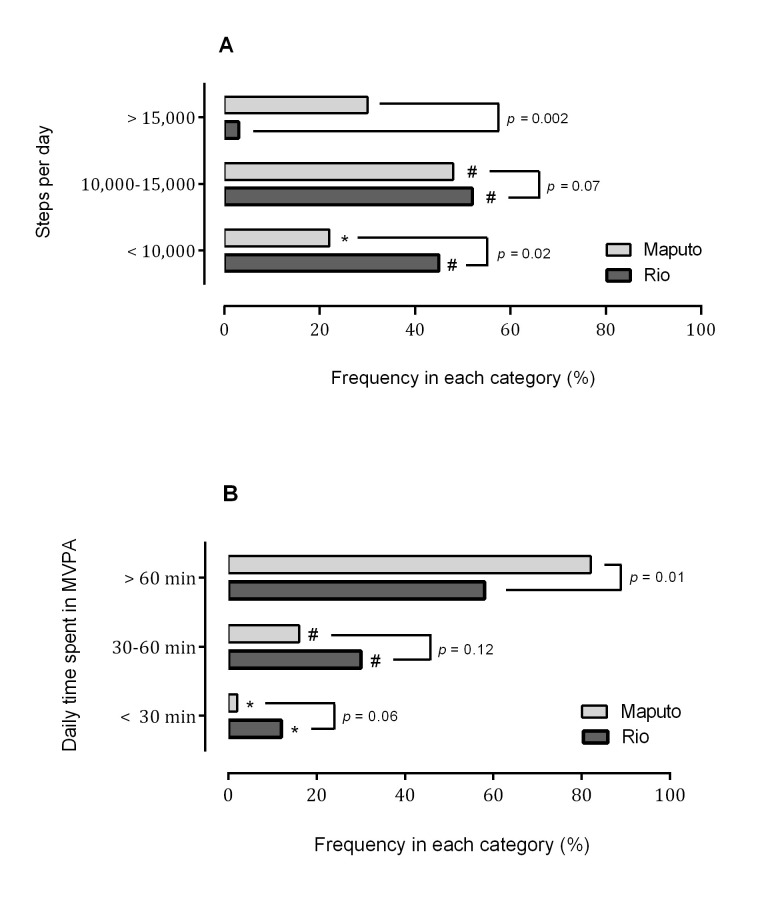
Relative frequency (%) of patients within PA categories determined over seven consecutive days based on: (A) Average steps per day; (B) Average time spent in MVPA. PA: physical activity; MVPA: moderate and vigorous physical activity. *: *p*<0.05 *vs.* 10,000-15,000 and >15,000 (A) or *vs.* 30-60 min and >60 min (B) #: *p*<0.05 *vs.* >15,000 (A) or *vs.* >60 min (B).

Figure 1 presents the proportion of individuals classified in each category of PA based on: a) average steps per day (Figure 1A); and b) average time per day spent in MVPA (Figure 1B). The percent frequency of participants classified as sedentary based on the number of steps per day was greater among Brazilian *vs.* Mozambican patients (45% *vs.* 22%, respectively). On the other hand, the proportion of individuals classified as active was much higher in Maputo (30%) than Rio (3%) (Figure 1A). Figure 1B shows that 82% of Mozambicans complied with the recommendations of at least one hour per day spent in MVPA, while only 58% of Brazilian patients performed this amount of PA. The proportion of patients performing 30- to 60 min/day and less than 30 min/day of MVPA were similar in the two countries. However, only 2% of Mozambican patients performed less than 30 min/day of MVPA *vs.* 12% of Brazilians.

## DISCUSSION

In the present study, PA level was objectively assessed in women of low SES living with HIV in two metropolis from low-income countries, in South America (Rio de Janeiro, Brazil) and sub-Saharan Africa (Maputo, Mozambique). The main results were as follows: a) The proportion of patients with overweight or obesity and prevalence of sedentary behavior were greater in Rio de Janeiro than Maputo; b) in general, daily PA was of light- to moderate intensity, while vigorous PA was almost non-existent, particularly among Brazilian patients; c) overall, higher levels of PA were found in Mozambican *vs.* Brazilian patients; d) regardless of differences in PA level, the average daily energy expenditure was similar across groups.

Over the last decades, funding for HIV/AIDS management increased in developing countries (32). Treatment with cART has become more widely available and counseling programs have expanded, which substantially improved the quality of life of PLHIV (33). The extended life expectancy due to cART is well-established. However, collateral effects of prolonged HIV infection and drug therapy represent a burden that must be counteracted. For instance, cART and inflammatory signaling pathways due to persistent immune activation predispose to increased metabolic and cardiovascular complications (4). In fact, cardiovascular disease seems to be the main cause of premature morbidity and mortality of patients under cART (3,4). Moreover, accumulated evidence shows PLHIV often exhibiting poor physical function (including sarcopenia) and low levels of PA (12,17,20,34), which compromise their work capacity, employment opportunities, and overall quality of life (5,8-10).

On the contrary, increased PA is acknowledged to counteract disability and improve mental, physical, and social outcomes of PLHIV (3,12,14,15,34). PA interventions applied to PLHIV with similar SES *vs*. those included in the present study, proved to be effective to improve their physical capacity and health. In Maputo, a prior study showed that women under cART increased their overall physical fitness in response to a 13-week aerobic and strength training program (35). In Rio de Janeiro, a structured multimodal program has been consistently shown to improve physical fitness, body image, and quality of life of low SES patients of both sexes, while preserving their immune function (36,37) and reducing the cardiovascular risk (38,39). However, strategies available in the literature are several, and even unorthodox approaches to increase the access of PLHIV to PA produced promising results. For instance, a recent controlled trial conducted in Italy, has shown that exercising with the support of a smartphone application was capable to improve physical fitness, metabolic, and psychological outcomes of middle-aged patients (40).

Despite these benefits, PLHIV usually exhibit poor levels of PA. Albeit the heterogeneity of available studies, data from extensive reviews (16,19) are consistent that in either developed or developing countries, the amount of daily activities performed by these patients with at least moderate intensity are insufficient. Overall, the average time spent in sedentary behaviors represented three-quarters of daily waking hours. The rest of awake time was found to be spent in light-intensity activities, which do not comply with the recommendations for the maintenance of physical function and reduction of cardiovascular risk (31). Actually, the mean time spent in moderate- to vigorous PA has been reported to range from 50- to 60 min per day in both young and older patients (16,17,19).

The major reason for this low adherence seems to be the over-congested public health systems, prioritizing the pharmacological treatment and neglecting other types of intervention. Concisely, almost all efforts focus on delivering antiretroviral drugs (32). Consequently, the access to facilities for structured or self-directed PA (as parks or sport fields) is limited, particularly in the case of PLHIV with low SES (41,42). Furthermore, SES seems to be the determinant of adherence to PA (21). Knowing more about the engagement of PLHIV of low SES in PA may help to devise effective public policies to increase their participation. This study adds to the current knowledge by providing an insight of PA patterns among female patients of low SES from socially vulnerable areas in two major cities in South America and sub-Saharan Africa, which have been neglected by prior research.

As previously mentioned, studies describing PA in developing countries are scarce. Vancampfort et al. (19,20) systematically reviewed quantitative studies on PA levels and correlates in PLHIV. In both reviews, only six studies were held in sub-Saharan Africa, including 1,015 patients aged 30 to 48 years (329 women) with sample sizes ranging from 42 to 407. A total of three studies were performed in South Africa, one in Ethiopia, one in Malawi, and one in Nigeria. Overall, the PA level in the observed populations was low. However, only two studies applied objective measures of PA; one in South Africa used pedometers (43) and a study in Ethiopia used ACL (44). Olsen et al. (44) observed 116 men (38±9 years) and 232 women (31±8 years), and reported 75-77% spending their time in sedentary behavior. Vigorous PA was found in 10% of males and 5% of females.

In the present study, 45% and 22% of women living in Rio de Janeiro and Maputo were sedentary, respectively. PA performed by patients was mostly of light and moderate intensity, while vigorous PA was practically inexistent (3-5 min of the day) and found in only 18% of participants in both cities. In one of the few studies objectively quantifying PA in an African country, Roos et al. (43) reported an average of 8103±833 steps/day in 42 patients aged 39±9 years (7 men) dwelling at Johannesburg (South Africa). This was slightly inferior, but comparable to our data regarding women from Maputo (12,853 steps/day) and Rio (10,099 steps/day).

The lack of experiments conducted in Africa also occurs in South America. Of the 45 studies included in the review published by Vancampfort et al. (19), only six were developed in this subcontinent, and all of them in Brazil. Samples ranged from 30 to 1240 individuals aged 20-59 years. In all studies, the PA information was obtained by means of self-reports (IPAQ or Baecke Questionnaire). Actually, we could not locate cross-sectional or cohort studies quantifying PA levels in PLHIV from South America by means of objective measurements. The possible exception was not designed to describe their PA, but to compare the energy expenditure assessed by doubly labeled water and ACL in individuals from Ribeirao Preto (SP, Brazil) (25). Patients with and without lipodystrophy exhibited similar energy expenditure at rest (1,433±196 *vs.* 1,510±203 kcal, respectively). The daily energy expenditure was also equivalent in both groups when assessed by ACL (2,560±458 *vs.* 2,594±456 kcal, respectively), or doubly labeled water (2,691±856 *vs.* 2,618±415 kcal, respectively). These values were greater than our data obtained in patients from Rio de Janeiro and Maputo (1,976 kcal and 1,933 kcal, respectively). However, the energy expenditure at rest in our samples was similar to results reported by Guimarães et al. (25) (1,520 kcal in Rio; 1,436 kcal in Maputo), which suggests differences across studies due to poorer PA levels in our patients.

Interestingly, although sedentary behavior was prevalent in samples from both Rio de Janeiro and Maputo, the proportion of patients complying with the minimum recommended PA for health promotion (at least 3 min/day of MVPA) was higher than values usually reported in developed countries, irrespective of the SES condition (16-19). A possible explanation for these findings, is that individuals with low SES usually engage more in work-related PA, followed by transport, and lastly in leisure (18), whereas the wealthier groups spend more time in leisure related PA (21). This discrepancy tends to be greater in low-income countries, since workers face transport difficulties and physical demands during work are usually high (41). Indeed, a high level of vulnerability within transport systems in both Rio de Janeiro and Maputo, including limited capacity, poor connections, and relatively high prices, are associated with low-income and negative socioeconomic indicators (45,46). The users usually have to walk long distances to reach public transportation facilities or their destinations. Unfortunately, we could not stratify the PA in the present study, and further research is warranted to confirm this premise.

As for the PA levels among populations other than PLHIV in these cities, there is a lack of research in Maputo. In Rio de Janeiro, we could find only a single study assessing PA by the International Physical Activity Questionnaire applied to 140 women from a low-income community (42). Approximately 55% of participants reported high levels of PA, mostly due to work and household activities. Although comparisons between studies may be compromised due to discrepancies in the assessment methods, they reinforce PA in this social group being mainly related to mandatory tasks. However, women of low SES living with HIV in our study seemed to be less active than their healthy counterparts.

Finally, it is remarkable that albeit the levels of MVPA had been ∼30% greater in patients from Maputo *vs.* Rio, they exhibited similar values for both daily energy expenditure and kcal spent in PA. The fact that Brazilian patients were heavier than those from Mozambique may have contributed to this result. Despite this, the time spent in PA *vs.* sedentary behavior was very short in the two samples, resulting in low values of average MET and daily energy expenditure. This is important, since the impact of PA upon some cardiovascular risk factors, as obesity, blood pressure, hyperglycemia, or hypercholesterolemia has been suggested to rely on the total amount of expended energy (31). Considering the cardiovascular risk being usually elevated in PLHIV, quantifying the overall PA in these patients would be useful to detect whether it is appropriate to improve their health outcomes.

Some limitations of the present study must be acknowledged. Firstly, the samples were relatively small and may not be representative of the two cities. Moreover, only women with low SES were included, and this limits the application of our findings to other groups. However, the objective assessment of PA in socially vulnerable areas in developing countries is always a difficult task. Although limited, our data provide original information to be further investigated in larger cohorts of PLHIV. Secondly, the lack of daily PA records precluded additional analysis on the type of PA performed by patients. This would be useful to determine the proportion of PA performed within labor, transport, or leisure tasks, in comparison with wealthier groups and low SES individuals living in developed countries.

Thus, the present study provides valuable information based on ACL about PA in Brazilian and Mozambican patients living with HIV, which is limited in those regions. In conclusion, overweight/obesity and sedentary behavior were more prevalent among HIV-infected women living in Maputo (Mozambique) than Rio de Janeiro (Brazil). Accordingly, levels of light and moderate- to vigorous PA were higher in Maputo *vs.* Rio. The recommended threshold of 60 min/day of moderate or vigorous PA was performed by approximately 80% of Mozambican *vs*. 60% of Brazilian patients. Overall, the amount of PA in PLHIV from both cities was low, so that the average daily energy expenditure was similar across the groups. At length, this might perhaps be insufficient to counteract cardiovascular risk or preserve their physical function. Additional research, objectively assessing PA of larger samples in different geographic areas of low-income countries in general, and Brazil and Mozambique in particular, are warranted to ratify these findings and determine the actual PA level among PLHIV with low SES more precisely.

## AUTHOR CONTRIBUTIONS

Mangona L was responsible for the conceptualization, data curation, formal analysis, investigation, methodology and manuscript original drafting. Brasil IA was responsible for the data curation, investigation, methodology manuscript original drafting. Borges JP was responsible for the data curation, formal analysis, investigation, resources, supervision and manuscript original drafting. Prista A was responsible for the conceptualization, formal analysis, methodology, investigation, supervision and manuscript original drafting, editing and review. Farinatti P was responsible for the conceptualization, data curation, formal analysis, investigation, methodology, project administration, resources, supervision, manuscript original drafting, editing and review.

## Figures and Tables

**Table 1:** Descriptive statistics of somatic and nutritional status (mean±SD) and frequency of individuals with overweight or obesity (absolute and relative).

	Rio de Janeiro (n=33)	Maputo (n=50)	*p-value*
Age	40.1±6.1	38.8±8.7	0.216
Height (m)	1.62±0.05	1.61±0.62	0.789
Body Mass (kg)	71.5±10.0	61.0±12.1	<0.001
BMI (kg/m^2^)	26.4±4.6	24.0±4.4	<0.001
Overweight (n, %)	13 (39.4%)	11 (22.0%)	0.089
Obesity (n, %)	6 (18.2%)	4 (8.0%)	0.167
Overweight+Obesity (n, %)	19 (57.6%)	15 (30.0%)	0.021

BMI: body mass index; *p-*values obtained from independent t-tests. Prevalence of individuals with overweight or obesity compared using percent values.

**Table 2:** Accelerometry data in patients from Rio (n=33) and Maputo (n=50) within seven consecutive days.

Variable	Rio de Janeiro Median (IQD)	Maputo Median (IQD)	Rio de Janeiro (mean rank)	Maputo (mean rank)	U	*p*
Kcal Activity (day)	456 (241)	497 (330)	36.8	45.5	995	0.144
Total Kcal (day)	1,976 (579)	1,933 (492)	43	41	788	0.731
Immobility (min/day)	554 (200)	569 (218)	41	42	969	0.180
Sedentary (min/day)	1,236 (142)	1,192 (135)	47	39	672	0.155
Light PA (min/day)	111 (56)	145 (51)	30	49.7	1.212	<0.001
Moderate PA (min/day)	59 (37)	84 (44)	32	48.9	1.169	0.001
Vigorous PA (min/day)	3 (4)	5 (4)	33.6	47.6	1.103	0.010
MVPA (min/day)	64 (36)	88 (46)	31.7	47.04	1.165	0.002
Step (day)	10,099 (3387)	12,853 (4795)	30	49.9	1.220	<0.001

BMI: body mass index; PA: physical activity; MVPA: moderate and vigorous physical activity. Kcal Activity corresponds to energy expenditure within movements performed in the three axes. Total kcal/day was estimated by means of the equation: average MET obtained during the day x (0.0035 x body mass (kg) x 1440) x 5 [Guimarães et al. (25)]. Immobility corresponds to periods during which the accelerometers did not record movements in any of the three axes.
